# Controlling Exciton/Exciton Recombination in 2-D Perovskite Using Exciton–Polariton Coupling

**DOI:** 10.1021/acs.jpclett.3c03452

**Published:** 2024-02-07

**Authors:** Rao Fei, Matthew P. Hautzinger, Aaron H. Rose, Yifan Dong, Ivan I. Smalyukh, Matthew C. Beard, Jao van de Lagemaat

**Affiliations:** † Chemistry and Nanoscience Center, National Renewable Energy Laboratory, Golden, Colorado 80401, United States; ‡ Materials Science and Engineering Program, University of Colorado, Boulder, Colorado 80301, United States; § Department of Physics, University of Colorado, Boulder, Colorado 80301, United States; ∥ International Institute for Sustainability with Knotted Chiral Meta Matter, Hiroshima University, Higashi Hiroshima, Hiroshima 730-0000, Japan; ⊥ Renewable and Sustainable Energy Institute, National Renewable Energy Laboratory and University of Colorado, Boulder, Colorado 80301, United States

## Abstract

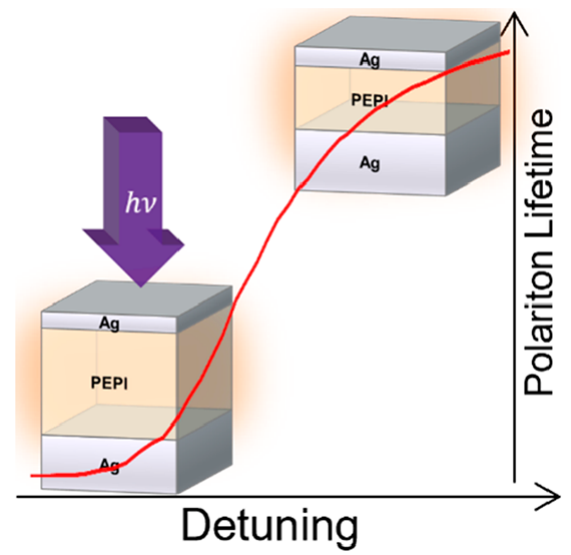

In this paper, we demonstrate that exciton/exciton annihilation in the 2D perovskite (PEA)_2_PbI_4_ (PEPI)—a major loss mechanism in solar cells and light-emitting diodes, can be controlled through coupling of excitons with cavity polaritons. We study the excited state dynamics using time-resolved transient absorption spectroscopy and show that the system can be tuned through a strong coupling regime by varying the cavity width through the PEPI layer thickness. Remarkably, strong coupling occurs even when the cavity quality factor remains poor, providing easy optical access. We demonstrate that the observed derivative-like transient absorption spectra can be modeled using a time-dependent Rabi splitting that occurs because of transient bleaching of the excitonic states. When PEPI is strongly coupled to the cavity, the exciton/exciton annihilation rate is suppressed by 1 order of magnitude. A model that relies on the partly photonic character of polaritons explains the results as a function of detuning.

Strong coupling between photonic and electronic states (e.g., excitons) typically occurs when the energy exchange between light and matter systems is faster than either of their decay rates.^1^ This phenomenon leads to the formation of hybrid states of light and matter, called polaritons. Coupling between a polariton photonic state and an exciton, for example, leads to the formation of two new exciton/polariton states referred to as the upper and lower polariton with the energy difference between the two polariton states termed Rabi splitting. These exciton/polariton states oscillate between the two original states at the Rabi frequency, typically on the femtosecond time scale.^2^ Such exciton/polaritons can be controlled by adjusting their relative amounts of photonic and excitonic character by tuning the relative energies and the coupling strength. Thus, by tuning the strong coupling, one can modify intrinsic properties of the material, such as the electronic energy level structure, energy transfer rates, and radiative and nonradiative recombination rates, and by extension control their photoinduced physics and even photochemical reactions.^3−5^

Optical cavities have been a powerful platform to demonstrate the quantum superposition of excitons and cavity modes.^6^ A Fabry–Pérot microcavity consists of two partially reflective mirrors and has been demonstrated for room-temperature polariton formation, manipulation, lasing, and condensation.^7^ The strength of the exciton/polariton coupling in such cavities can be controlled by controlling the quality factor, the relative layer thicknesses inside the cavity, and other aspects such as temperature and polarization.^8,9^

2D transition metal dichalcogenides (2D-TMDCs) with strong light absorption and large exciton binding energies have been proposed and some have been demonstrated to offer a distinctive platform to achieve room temperature strong coupling.^10^ Similarly, 2D metal-halide perovskites as another class of 2D semiconductors exhibit intriguing optoelectronic properties, including high optical absorption, large and tunable exciton binding energies,^11^ and high carrier mobilities, while showcasing a unique set of excitonic effects that becomes more pronounced as they transition from the bulk to the confined multiple quantum wells structure of the 2D configuration (e.g., metal-halide layer thickness).^12^ This scaling results in a larger bandgap and exciton binding energy, rendering them particularly attractive for room temperature excitonic devices.^13,14^

Several studies on a variety of material systems reported polariton lifetimes matching the order of magnitude of the cavity photon lifetimes^15,16^ in the strong coupling regime, while other works showed that polaritons can survive much longer than cavity photons and even longer than the uncoupled excitons.^17−19^ The explanation for these observations often focuses around the more delocalized nature of the polariton states giving rise even to ballistic transport^20^ or coupling of the excitations to uncoupled “dark states”^21^ but no clear unifying explanation. To gain further insight, we introduce a promising avenue in a 2D perovskite system, taking advantage of its strong excitonic character.

Here, we demonstrate ultrastrong coupling of the 2D perovskite (PEA)_2_PbI_4_ (PEPI) with the first order cavity mode of a silver Fabry–Pérot microcavity. We establish a system with different cavity thicknesses and observe a record room-temperature Rabi splitting for PEPI of 265 meV. By means of ultrafast pump probe spectroscopy, the kinetics probed for the lower polariton energies in our system reveal a longer lifetime of the excited states in the strongly coupled system and demonstrate that the excited state kinetics can be controlled by detuning (i.e., adjustment of the resonance frequency of the microcavity), which is achieved by varying the thickness of the PEPI layer. The results are compared to a simple model that uses the Hopfield coefficients determined from the steady-state absorbance spectra to estimate the photonic vs excitonic character of the excited state. Such control of exciton–exciton annihilation, which is a major loss mechanism in lasers and other optoelectronic devices, could allow for much higher efficiency in optoelectronic elements based on systems like PEPI.

## Tuning Coupling Strength in a Low Quality Cavity

**Figure 1:**
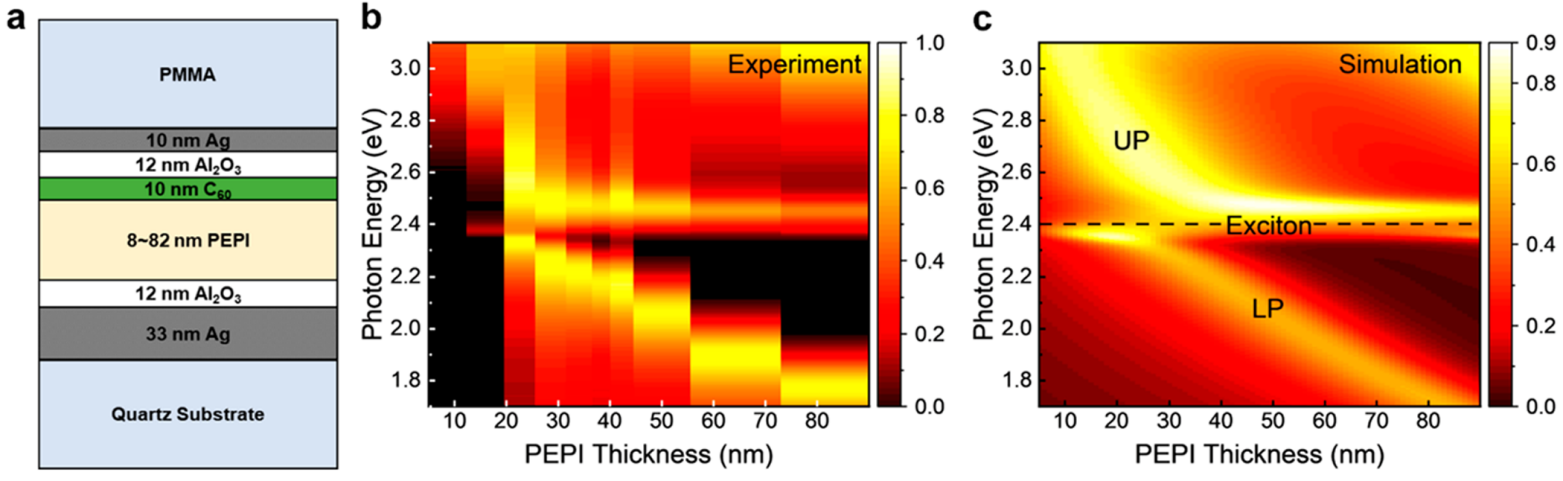
Microcavity system–simulation and experimental dispersions. (a) Schematic drawing of the microcavity system. (b,c) Experimental and simulated dispersions of the strongly coupled cavity system. The absorptance is plotted on the 2D surface of the photon energy versus PEPI thickness. The room temperature absorptance of 10 samples (b) is calculated from the reflectance and transmittance measured with ellipsometry, while the actual PEPI thicknesses are plotted at the center value of each thickness bar. The theoretical thickness dispersion (c) is computed with COMSOL using the refractive indices obtained at room temperature (see Experimental Methods).

Figure 1a shows a schematic of the experimental strong coupling system. Ellipsometry data are analyzed to obtain the thickness of each deposited layer as we build the system with layer-by-layer deposition techniques (see Experimental Methods in the Supporting Information).

Strong coupling can be observed in the dispersion plots as two distinct coupled modes exhibiting anticrossing behavior at the bare exciton energy. Our theoretical and experimental dispersion plots exhibit a high degree of agreement (Figure 1b,c); both show the two branches of the coupled modes with a splitting at the exciton energy *E*_PEPI_ = 2.40 eV. The modes above and below this energy are the upper polariton (UP) and the lower polariton (LP) branches, respectively.

The Rabi splitting is defined as the minimum energy separation between the UP and LP peaks, which occurs when the coupling is maximized by virtue of the thickness being ideally tuned and is proportional to the coupling strength. Away from this condition, the cavity is detuned, and therefore, the thickness of the active layer allows for a simple way to control the amount of coupling between cavity polaritons and excitonic states. Due to the fact that varying the PEPI thickness also changes the number of oscillators in eq 1, formally the Rabi splitting depends on the PEPI thickness therefore we only list the Rabi splitting where the energy separation is minimal. This does not affect any of the analysis of the transient data below. The Rabi splitting is 273 meV for 34 nm of PEPI thickness in the simulation, and we find 265 ± 5 meV at 35 nm in the experiment with a relative coupling strength (η) of 11%. Systems with Rabi splitting greater than 10% of the exciton energy can be defined as ultrastrong coupling systems.^22^ Typically, achieving such coupling strength demands a high-quality factor of the cavity, leading to limited accessibility to the system (i.e., no light leaks out of the cavity). However, here, we demonstrate how, through leveraging nanoscale fabrication and the strong excitonic nature of PEPI, an accessible ultrastrong coupling system can be realized and characterized using transient absorption spectroscopy in transmission mode.

In studies of the strong coupling between cavities and semiconductors, Fabry–Pérot cavities and Bragg mirrors are frequently employed, and they typically have a quality factor on the order of 100,^23−25^ implying limited access to the cavity, primarily limited to optical methods. The quality factor of our structure is calculated as the ratio of its resonance frequency and the full-width at half-maximum of the resonance peak, from the simulation of the bare cavity absorption spectra, giving *Q* = 3.59 with the 10 nm Ag/12 nm Al_2_O_3_/10 nm C_60_/34 nm PEPI/12 nm Al_2_O_3_/33 nm Ag structure. The low quality factor agrees with our simulation and experimental angular dispersion plots (Figure S2c,d), which show little detuning with changing the incident angle, and ensures optical accessibility to the cavity, while maintaining the ultrastrong coupling strength. This outcome is attributed to the controlled thicknesses of the layered structure within the first order cavity mode during fabrication (Figure S2a and b) and the fact that spin-coated 2D perovskite layers remain highly axially oriented with the PbI_4_ planes perpendicular to the substrate (Figure S1). Remarkably, the excitonic resonances also remain narrow even with spin-coating/solution deposition methods employed. However, we were not able to observe photoluminescence from these cavities due to the low photoluminescence efficiency and the low outcoupling efficiency. In the future, the cavities can probably be tuned to observe luminescence from the lower polariton as would be expected.

## Exciton/Polariton Dynamics

**Figure 2:**
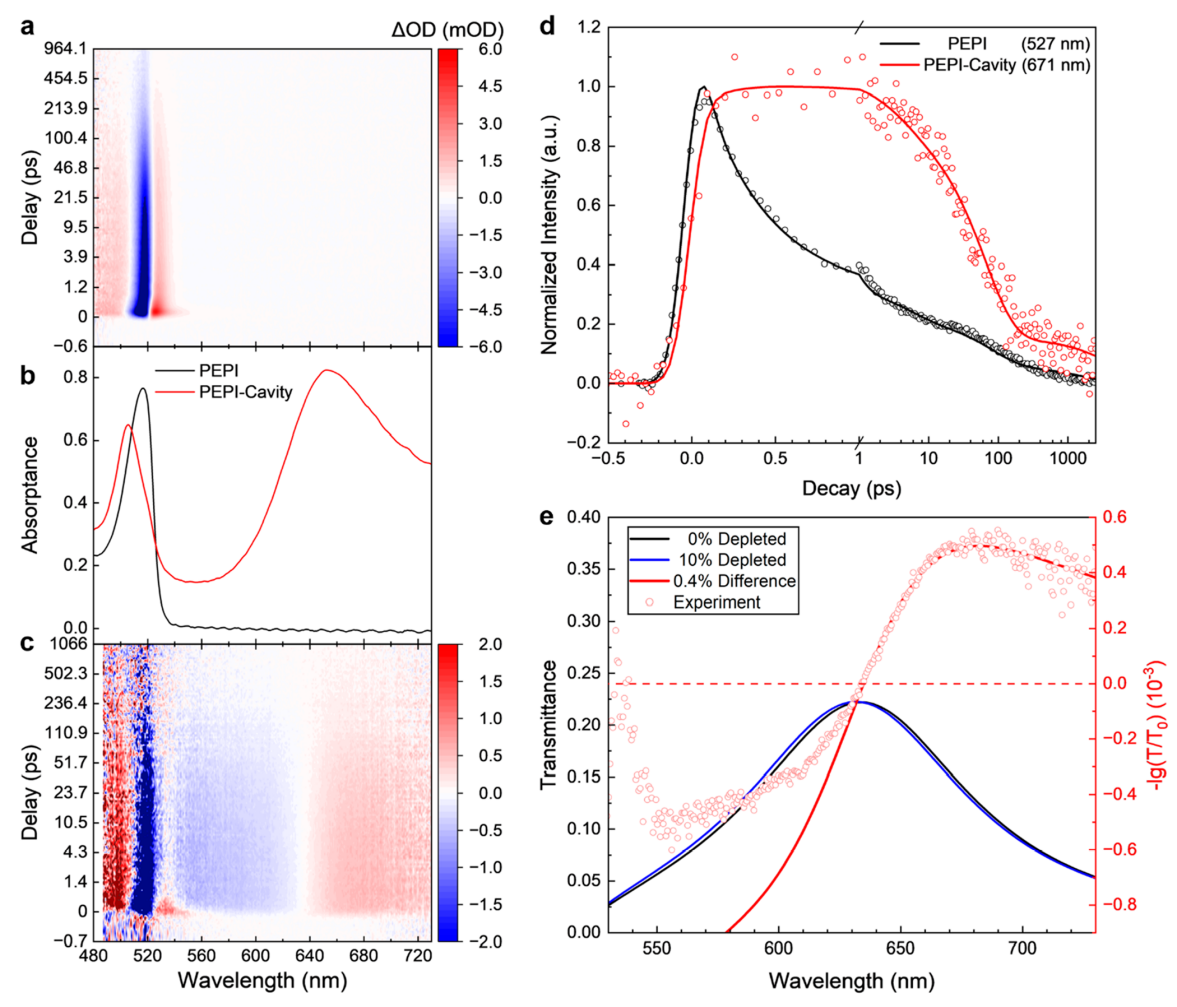
Photophysical characterization of the PEPI-microcavity system. (a,c) Transient absorption spectra of a bare PEPI film (a) and a 64 nm PEPI in a microcavity (c), both pumped at 405 nm. (b) Steady-state absorption spectra of the same PEPI and PEPI-cavity samples measured with ellipsometry. (d) Decay traces of the bare PEPI and the cavity system, measured at 527 and 671 nm, respectively. The positive component in the derivative-like feature is chosen to avoid overlapping with the signal from uncoupled PEPI. (e) Prediction of the derivative-like features at the LP wavelengths using a single oscillator model representing the PEPI exciton. The transmittance spectrum at 10% depletion of the oscillator is plotted to show the shifting of the LP peak, while the actual depletion here is fitted to be 0.4%; experimental data are taken from the same microcavity sample and averaged from 0.7 to 3.8 ps.

Figure 2a shows the room temperature broadband transient absorption spectra for a PEPI thin film excited using a 405 nm pump (3.06 eV), which is at a much higher energy than the exciton and the LP resonances, and we chose this wavelength to be able to excite the bare layers and the cavity samples at the same energy. For the cavity samples, it would also be interesting to pump at the UP energy, however this was not attempted for this study as it cannot be directly compared. We observe a strong excitonic photobleaching at 517 nm and two photoinduced absorption peaks at 493 and 527 nm, which closely matches the observations in the existing literature and can be associated with a combination of state filling and bandgap renormalization.^12,26^ Their decay dynamics (at 493, 517, and 527 nm) are also found to be closely proportional to each other.^26^

In contrast, the transient absorption spectra for the microcavity samples (a representative detuned microcavity in Figure 2c) show derivative-like features at the LP resonance energies in addition to the three peaks arising from the exciton of PEPI molecules as discussed above for the uncoupled control samples but is now better described as the upper polariton that is only slightly shifted in energy and has possibly uncoupled PEPI molecules mixed in. The center wavelengths of the derivative features show a strong concurrence with the peak positions of the LP from the linear absorption spectra in Figure 2b (also see Figure S3). The trace at LP energy from the PEPI-cavity system as well as at the upper polariton and exciton energies shows significantly slower excitation and decay as compared to the bare PEPI control (Figure 2d).

In strongly coupled cavity systems, the Rabi splitting energy is proportional to the square root of the oscillator strength and number of oscillators:^27^
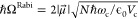
1where μ⃗ is the transition dipole moment, ℏω_c_ is the cavity mode at resonance with the exciton, ϵ_0_ is the vacuum permittivity, *V*_c_ is the volume of the cavity, and *N* is the number of oscillators that are strongly coupled to the cavity. When the system is optically pumped at 3.06 eV, the oscillator strength is reduced and the Rabi splitting decreases, causing shifts of the UP and LP peaks and resulting in derivative-like features at the UP and LP positions in the transient spectrum. To model this, we modeled the permittivity of PEPI using a single Lorentzian oscillator representing the excitonic transition at 517 nm (2.4 eV; Figure S1) and employed that in our simulated structure. By decreasing the amplitude of the oscillator, essentially representing the depletion of available transitions or the excitation of the system by the optical pump excitation, we obtain a differential spectrum that predicts the shape of the transient spectrum (Figure 2e) very accurately on the low energy side but misses the high energy side as there are more transitions in the PEPI layer at higher energies that are not captured in the simplified model of a single Lorentzian oscillator. In the obtained transient spectrum, the percentage of depletion mostly affects the amplitude of the derivative-like features in linear fashion; at the same time it causes a shift that is not observable at our experimental wavelength resolution and pump fluences. We find that the spectrum is best described using an estimated 0.4% of the excitonic states bleached by the optical pump pulse. Here, we focus on LP, but the same analysis at shorter wavelengths shows the same behavior (Figure S4). This then indicates that the decay trace of the derivative features represents the dynamics of the entire strongly coupled system and not of a single UP/LP state because the derivative feature is mostly caused by the pump induced change in Rabi splitting, and the pump-induced population of the polariton states only has a minor effect on the transient spectra.

Carrier dynamics in semiconductors are typically described by the following equation:
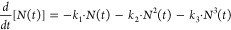
2where *N*(*t*) is the carrier density at a pump–probe delay of *t*, and *k*_1_, *k*_2_, and *k*_3_ are the monomolecular, bimolecular, and trimolecular recombination rate constants, respectively.^12^ The total carrier density *N*(0) is calculated as the total number of photons absorbed by the cavity (see the detailed calculation in Note 1 in the Supporting Information).

**Figure 3:**
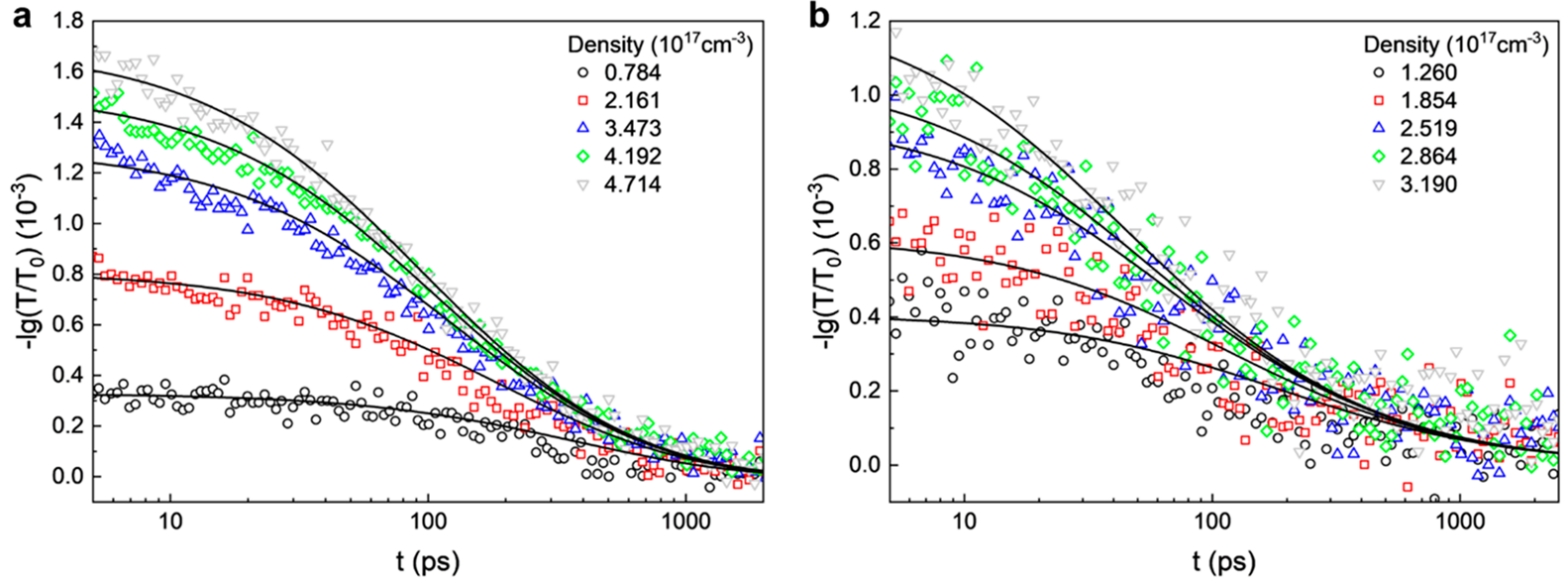
Global fitting of carrier dynamics. (a) In a bare PEPI film. (b) In a PEPI-microcavity system with 47 nm PEPI.

The exciton decay traces measured under different fluences are globally fitted for each sample using eq 2 at each pump intensity simultaneously so that one set of *k*_1_, *k*_2_, and *k*_3_ parameters is obtained for each sample consistent with the traces at all pump intensities. Since the thermalization process is not described in this equation, we do not include the first 5 ps in our modeling (Figure 3a). The LP decay traces are fitted including the first 5 ps as the derivative features are observed to happen only after the thermalization (Figure 3b). In the strong coupling case, *k*_1_ becomes smaller than *k*_2_, *k*_2_ decreases by a factor of 2, and the clarity of *k*_3_ necessitates a further increase in fluence. Results from different PEPI thicknesses are shown in Table 1.

**Table 1:** Measured Recombination Rates for Different PEPI Thickness

PEPI thickness (nm)	trace picked at (nm)	*k*_1_ (ns^–1^)	*k*_2_ (10^–20^ cm^3^·ps^–1^)	*k*_3_ (10^–37^ cm^6^·ps^–1^)
41 (control)	527	1.04 ± 0.09	9.27 ± 0.24	0 ± 1.1
64 (cavity)	671	0 ± 0.38	5.02 ± 0.60	0.99 ± 0.39
47 (cavity)	620	0 ± 0.30	4.57 ± 0.42	1.23 ± 0.29
38 (cavity)	588	0 ± 0.28	4.01 ± 0.38	2.62 ± 0.21
35 (cavity)	573	0 ± 0.20	0.35 ± 0.07	0.38 ± 0.01
28 (cavity)	557	0 ± 0.33	1.26 ± 0.30	2.27 ± 0.14

The monomolecular rate *k*_1_ is commonly assigned to radiative and nonradiative traps limited recombination that can reflect the sample quality. The fact that *k*_1_ decreases in the cavity is likely due to the use of C60 as passivation layer, or indicates that the interactions between polaritons and defects are typically lower than those between excitons and defects, where one is photonic, and the other is excitonic.

**Figure 4:**
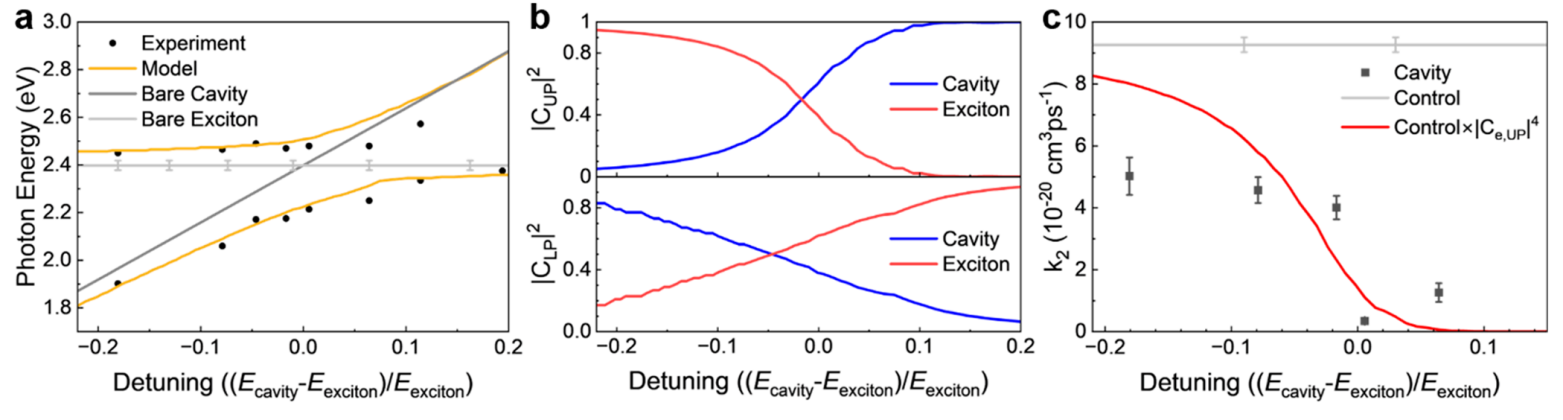
Determining hybridization coefficients and predicted trend of the biexciton recombination rates. (a) Simulation (yellow line) and experimental (black points) peak positions of the thickness dispersions. The bare cavity mode is modeled with a fictitious lossless material, discussed in Figure S1. (b) Hopfield coefficients for different PEPI thicknesses in the UP state are calculated from the thickness dispersion (a). (c) Bimolecular recombination rates *k*_2_ for bare PEPI, microcavities with different PEPI thicknesses for which transient data were obtained, along with the corresponding error bars obtained from the fitting, and a prediction (red line) of *k*_2_ assuming the system is purely in the UP state.

The bimolecular rate *k*_2_ is usually assigned to exciton–exciton annihilation. Compared to that of the bare exciton of PEPI, *k*_2_ significantly decreases in the cavities, as shown in Figure 4c, with a larger decrease for stronger coupling (smaller detuning, thinner films). This results from the nature of polaritons embodying the characteristics of both light and matter in a partial manner and is consistent with earlier observations on the 2D semiconductor MoS_2_.^28^

Here, we use a semiclassical coupled harmonic oscillator model to quantify the strength of the coupling and explain how it modifies *k*_2_. The Hamiltonian of the system can be written as
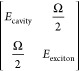
3where *E*_cavity_ is the bare cavity energy, *E*_exciton_ is the bare exciton energy, modeled with the existence of the surrounding dielectric medium (Figure 4a), and Ω is the Rabi splitting energy at zero detuning. The Hamiltonian is solved and its eigenvalues yield the UP and LP modes. We calculate the eigenvectors of the Hamiltonian, to show how the cavity and the exciton hybridize into polaritons:
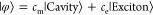
4

The Hopfield coefficients |*c*_*i*_|^2^ shown in Figure 4b represent the weighting of the hybrid modes. In our PEPI-microcavity system, the contribution of the PEPI exciton to the UP decreases with increasing detuning (decreasing PEPI thickness), showing that the UP is purely photonic at high positive detuning (PEPI thicknesses below 20 nm), and it evolves into purely excitonic at high negative detuning. The LP shows opposite contributions. At the point of zero detuning, both UP and LP show equal weighting from the photonic and excitonic modes.

A simple model of the polariton indicates that |*c*_m_|^2^ can be interpreted as the probability of being a photon and |*c*_e_|^2^ as the probability of being an exciton. If we assume that recombination only occurs while the polariton is an “exciton,” then the observed bimolecular recombination rate *k*_2_ of the coupled system is
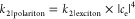
5assuming the annihilation only happens when the two particles are both excitons. As we pump the system at a much higher energy (3.06 eV), we expect the carriers to be mostly in the UP state. A prediction of the bimolecular recombination rates is plotted in Figure 4c. Assuming the entire system comes to the UP state after the excitation, the bimolecular rate follows the shape of |*c*_e,UP_|^4^. While this is a first order approximation based on the simplest model, it surprisingly predicts the decreasing trend of *k*_2_ with increasing detuning. The difference in our experimental *k*_2_ data can be due to a small portion of the carriers in the LP state, which could potentially yield a method to measure the UP/LP ratio.

In summary, our experiments show good tunability of the PEPI-microcavity system into an ultrastrong coupling regime (265 meV, η = 11.0%) by adjusting the cavity width. The observed derivative-like transient absorption spectra can be effectively described using a time-dependent Rabi splitting calculated from a single Lorentzian approximation of the optical constants of PEPI. When strongly coupled to the cavity modes, the exciton/exciton annihilation rates in PEPI decrease due to the formation of polaritons with partly photonic character. In this way, the exciton lifetimes and related processes in semiconductors can be further manipulated. These findings contribute to a deeper understanding of the fundamental phenomena in light-matter strong coupling and may enable enhanced control over energy dissipation in applications such as solar cells and light-emitting diodes.

## Data Availability

The data that support the findings of this study are available within the paper and its Supporting Information. Source data are available from the corresponding author upon reasonable request.
