# Supplementary material for: Controlling Exciton/Exciton Recombination in 2-D Perovskite Using Exciton–Polariton Coupling
Source: J Phys Chem Lett. 2024 Feb 7;15(6):1748–54. doi: 10.1021/acs.jpclett.3c03452 (PMC10875656; doi:10.1021/acs.jpclett.3c03452)
Supplement: Supplementary file 1 — jz3c03452_si_001.pdf [file jz3c03452_si_001.pdf]

Supporting Information

Controlling Exciton/Exciton Recombination in 2-D Perovskite using Exciton–Polariton Coupling

Rao Fei[1,2], Matthew P. Hautzinger[1], Aaron H. Rose[1], Yifan Dong[1], Ivan I. Smalyukh[2,3,4,5], Matthew C. Beard[1,5], Jao van de Lagemaat[1,2,5]

[1]Chemistry and Nanoscience Center, National Renewable Energy Laboratory, Golden, CO, USA.

[2]Materials Science and Engineering Program, University of Colorado, Boulder, CO, USA.

[3]Department of Physics, University of Colorado, Boulder, CO, USA.

[4]International Institute for Sustainability with Knotted Chiral Meta Matter, Hiroshima University, Higashi Hiroshima, Hiroshima, Japan

[5]Renewable and Sustainable Energy Institute, National Renewable Energy Laboratory and University of Colorado, Boulder, CO, USA

Corresponding Author

Jao van de Lagemaat[1,2,5] Jao.Vandelagemaat@nrel.gov

Experimental Methods

Layer-by-layer Deposition of PEPI in Microcavity

Fused quartz substrates were purchased from Advalue Technology (stock number FQ-S-001). The substrates were cleaned by sonicating in acetone then isopropyl alcohol for 15 min each, dried with a nitrogen spray gun, and stored in a nitrogen dry box before use.

Ag deposition was performed by electron beam deposition of a very thin layer of Ti followed by the Ag. The thicknesses of Ti/Ag for all sample were verified by ellipsometry, are around 1 and 33 nm respectively.

Atomic layer deposition (ALD) of Al_2O_3 was then performed under 90°C, to obtain an isolation layer between Ag and the next PEPI layer, which prevents the chemical reaction between Ag and PEPI and also helps prevent any possible charge transfer between the layers. The thickness of Al_2O_3 was confirmed by ellipsometry to be around 12 nm, with the stacked layers and blank witness samples (based on silicon wafers). The Al_2O_3 coated substrates were stored in a nitrogen dry box and then coated by the next layer of PEPI within several hours.

The stock solution needed for spin-coating PEPI films was obtained as follows. 230 mg (0.5 mmol) of PbI_2 (99.9985%, Sigma Aldrich) and 249 mg (1 mmol) of phenylethylammonium iodide (PEAI, 98%, Sigma Aldrich) were dissolved in 1 mL of N,N-Dimethylformamide (DMF, Sigma Aldrich) and filtered with a 0.22 μm PTFE syringe filter to make a 0.5 M stock solution. This stock solution was then serial diluted to obtain the desired concentrations (0.03-0.17 M) for different thicknesses of PEPI. The solution was then spin-coated onto previous substrates for 20 s at 4000 RPM. The films were then annealed at 100 °C for 10 min.

To build the rest of layers making up the microcavity, 10 nm of C_{60} was deposited on PEPI by thermal evaporation to protect PEPI from the deposition of subsequent layers, followed by another ALD of 12 nm Al_2O_3, then the electron beam deposition of 10 nm Ag. Finally, the microcavities were sealed by spin-coating polymethyl methacrylate from a chlorobenzene solution on the top, to prevent/slow down any oxidation of the samples.

The control sample without silver mirrors was fabricated similarly with ALD-Al_2O_3 / spin-coated-PEPI on the quartz substrate.

Ellipsometry for Thicknesses and Refractive Indices

The refractive index of PEPI on Al_2O_3 on Ag on fused silica substrate was modeled from ellipsometry data. Ellipsometry data was collected using a J.A. Woollam M-2000 ellipsometer. The data was modeled in J.A. Woollam WVASE software. The first Ag layer on quartz was modeled as: fused silica substrate / 1 nm Ti / 33 nm Ag / 0.25 nm Ag_2S / 12 nm Al_2O_3. The thickness results compare well with the witness samples, that are fused silica substrate / 1 nm Ti / 33 nm Ag, and silicon wafer substrate / 12 nm Al_2O_3. The fused silica and Al_2O_3 refractive indices were taken from the WVASE library where Al_2O_3 was from Lichtenstein, Ti and Ag were taken from Palik,[1,2] and Ag_2S from Bennett.[3] This model was then extended to model the PEPI on top of them. The parameters for PEPI were fit along with thickness, while other model parameters determined in the previous step were kept constant. The PEPI film used for this fitting resulted in a thickness of 105 nm, along with a uniaxial model of its optical parameters (Figure S1).

Based on the refractive index of PEPI obtained as above, the microcavity samples were modeled as: quartz / Ti / Ag / Ag_2S / Al_2O_3 / PEPI / C_{60} / Al_2O_3 / Ag, where the quartz refractive index was taken from

Palik,[4] the C_{60} was modeled with a quartz substrate / 10 nm C_{60} sample and was found to closely match the values from Aboura.[5] The thicknesses of C_{60}, Al_2O_3 and Ag on the top were confirmed with witness samples as 10, 12 and 10 nm respectively in average.

Theoretical Calculations with COMSOL

Electromagnetic simulations were performed with the COMSOL Multiphysics® software (v. 5.6) to obtain the absorption spectra. The cavity structure was built from the average thicknesses among all the samples, as 10 nm incident Ag / 12 nm Al_2O_3 / 10 nm C_{60} / sweeping thickness of PEPI / 12 nm Al_2O_3 / 33 nm output Ag. The refractive index of PEPI was measured by ellipsometry, and the indices for Ag (10-30 nm), Al_2O_3, C_{60} were the same as used in ellipsometry fitting.

Ultrafast Transient Absorption Spectroscopy

Transient absorption spectroscopy was conducted with a Helios Fire spectrometer to characterize the dynamics of the system. The pump and probe pulses were generated by a PHAROS laser system. The pump laser was generated from a Yb:KGW medium and tuned to 405 nm with an Orpheus optical parametric amplifier at 1 kHz, and was chopped into 0.5 kHz to allow collection of the "pump off" spectra. The probe laser was originally generated from the same medium onto a thin sapphire window to impart white light (460-800 nm) at 1 kHz. The pump and probe beams were spatially overlapped on the samples. The excitation of the cavity samples was done from the thinner silver side. The pump fluences were maintained at $\leq 2.1\ \mu J/cm^2$ for bimolecular recombination rate measurements.

Global Fitting Method

The dynamics of the LP and exciton signals are fitted to the ordinary differential equation in equation (2) using the global fitting routines available within Igor Pro 9.01 which implements a Levenberg-Marquardt minimization of the square deviations.

Note 1: Carrier concentration calculation

The absorptance for microcavity and control samples were measured by ellipsometry at the pump wavelength 405 nm. The total carrier density is calculated as the pump energy divided by the beam size and the length of path, which is the thickness of PEPI in our system. For such a typical 5.3 nJ pump energy (an example from the control dataset), the total carrier density is

$$N(0) = \frac{5.3\ nJ \times 0.4668}{\pi \times 0.02557\ cm \times 0.04412\ cm \times 3.061\ eV \times 41\ nm} = 3.473 \times 10^{17} cm^{-3}.$$

Note 2: Photon lifetime in Fabry–Pérot cavities

In Fabry–Pérot cavities, the light beam bounces back and forth between the two reflective surfaces. The lifetime of a photon in a bare cavity is typically very short even for high Q cavities, it can be calculated as $\tau_p = \frac{2nL/c}{1 - R_1R_2}$, where n is the refractive index, L is the cavity width, c is the speed of light, R_1 and R_2 are the reflectivity of the two surfaces. With $n = 1.937$, $L \approx 100$ nm, $R_1R_2 \approx 0.52$, we get $\tau_p \approx 2.69$ fs. Even if we assume higher reflectivity from the surfaces, this effect is smaller than the pulse width used in our experiments (~50 fs) and does not affect the analysis of the rate constants.

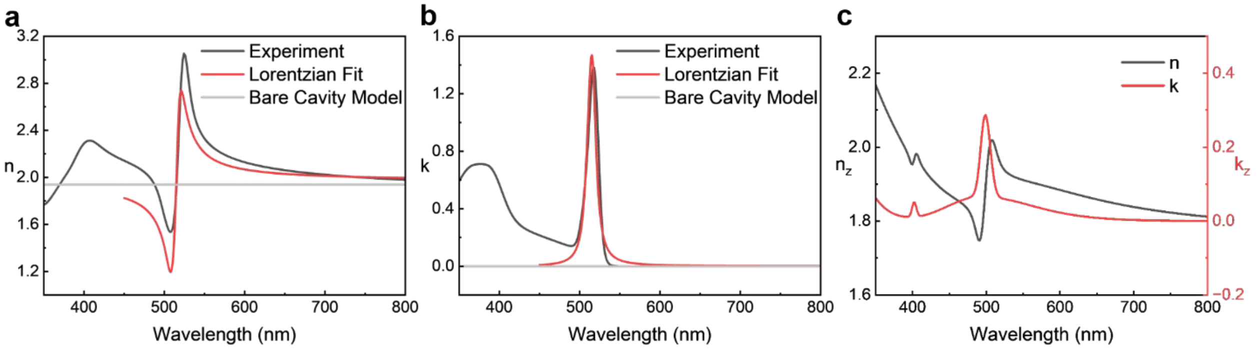

Figure S1 Refractive indices of 2D PEPI.

The 2D PEPI has 2 optic axes, the real and imaginary parts of the in-plane index are plotted in (a) and (b) respectively, and (c) shows the out-of-plane index. The in-plane oscillator strength is much higher than that in the out-of-plane direction, suggesting the PbI_4 plane is oriented perpendicular to the substrate. The black lines of (a) and (b), and both lines in (c) show the refractive index of PEPI measured by ellipsometry, which are used in the simulation of the dispersion plots and fitting the thicknesses. The red lines in (a) and (b) are fitting the refractive index into a single Lorentzian oscillator and are then used in simulation of the transient spectrum. The permittivity is fitted using the following equation:

$$\begin{cases} \varepsilon_1 = \varepsilon_{bg} + \frac{A(\nu_0^2 - \nu^2)}{(\nu_0^2 - \nu^2)^2 + (\Gamma\nu)^2} \\ \varepsilon_2 = \frac{A\Gamma\nu}{(\nu_0^2 - \nu^2)^2 + (\Gamma\nu)^2} \end{cases} \quad \text{(S1)},$$

where $\varepsilon_{bg} = 3.751$ is the background permittivity, $A = 4.565 \times 10^{28}(\text{Hz}^2)$ is the amplitude of the single Lorentzian oscillator, $\nu_0 = 5.800 \times 10^{14}(\text{Hz})$ the resonant frequency, and $\Gamma = 1.208 \times 10^{13}(\text{Hz})$ the full

linewidth. When modeling the bare cavity modes, we use a fictitious lossless 2D material with n = 1.937, which comes from the Lorentzian fitting, and k = 0 as the in-plane index, shown as the grey lines, while the out-of-plane index is kept the same as PEPI's, to approximate the dielectric environment of uncoupled bare cavity.

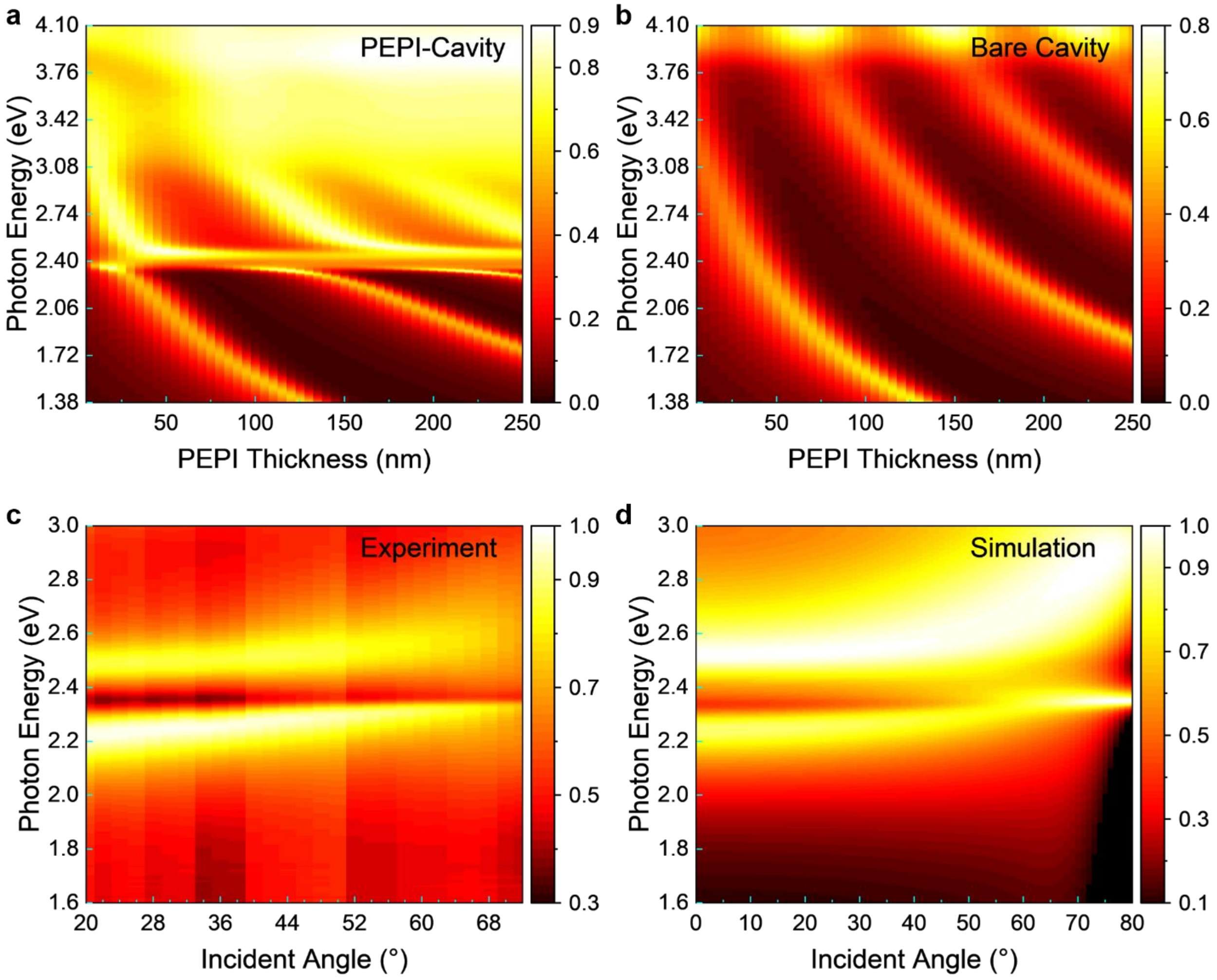

Figure S2 Dispersion analysis of strongly coupled PEPI-microcavity system.

a,**b**, Simulated dispersions of the strongly coupled PEPI-microcavity system (**a**), and that of bare cavity system (**b**), absorptance is plotted on the 2D surface of photon energy vs PEPI thickness. For bare cavity simulations, we use the fictitious lossless PEPI described in Figure S1. These dispersion plots show the positions for the first, second and a head of the third order cavity modes. In this study, the samples are constructed with PEPI thicknesses within the range of the first order cavity mode. This helps reduce the interference from other cavity modes, and increase the coupling strength. **c**,**d**, Experimental (**c**) and simulated (**d**) angular dispersion of a 35 nm PEPI coupled to microcavity, absorptance is plotted on the 2D surface of photon energy vs the incidence angle. The UP and LP at 2.52 eV and 2.24 eV show little detuning with the change of incident angle, due to the low quality factor of the cavity.

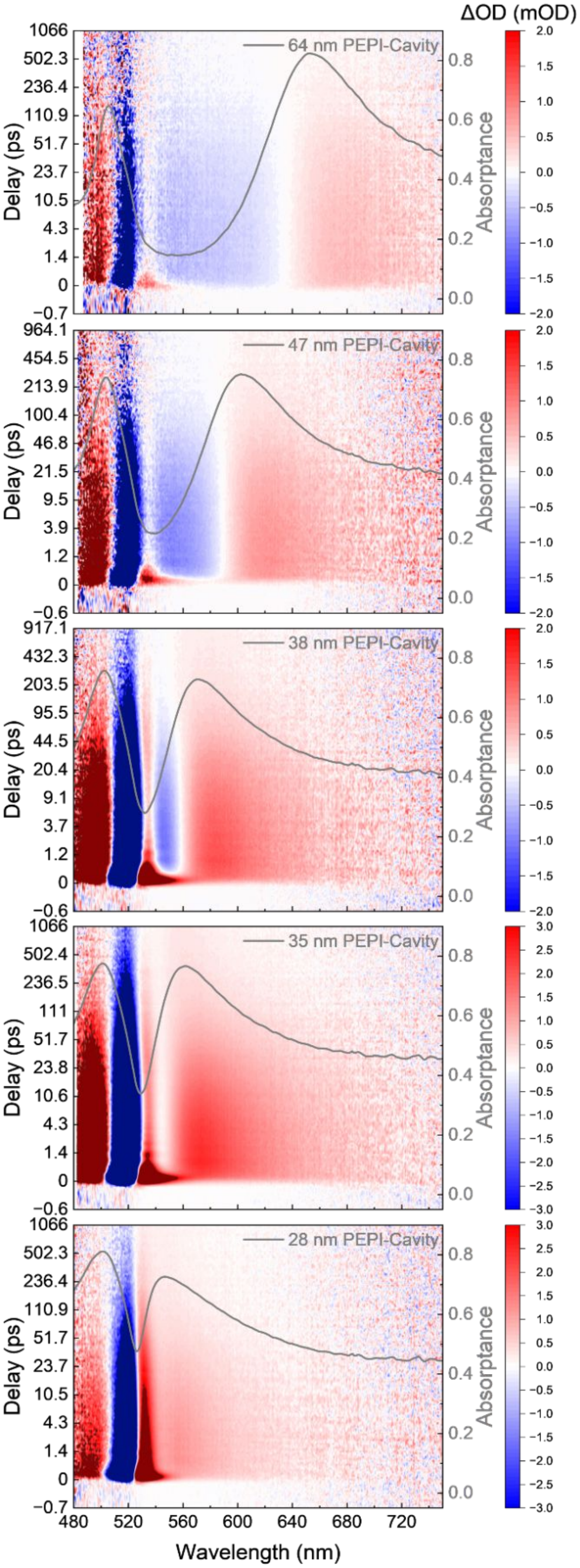

ΔOD (mOD)
64 nm PEPI-Cavity
47 nm PEPI-Cavity
38 nm PEPI-Cavity
35 nm PEPI-Cavity
28 nm PEPI-Cavity
Delay (ps)
Absorptance
Wavelength (nm)

Figure S3 TA spectra of PEPI-microcavity samples with different PEPI thicknesses.

The derivative features align well with the wavelengths of LP states measured by ellipsometry (black line), which shift toward shorter wavelength with decreasing cavity thickness.

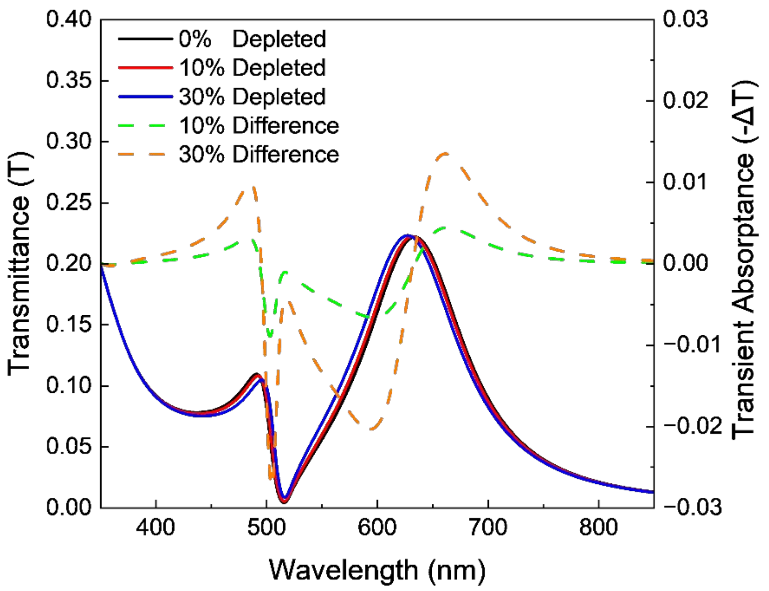

Figure S4 Transient spectra modeled as decreased oscillating strength.

The transmission spectra are simulated as a 64 nm PEPI film with refractive index fitted in Figure S1, sandwiched in the microcavity, where the depletion of the system is the portion reduced in the Lorentzian amplitude. While in experiments the depletion of the oscillator would not be over 1%, the 10% and 30% depleted transmittance spectra clearly show the shifting polariton peaks. With the excitation of the system, the Rabi splitting decreases, resulting in 2 derivatives at the polariton wavelengths. Increasing the depletion would cause a linear increase in the amplitudes and a negligible shift of the peak positions in the transient derivative-like features.

References

(1) Lynch, D. W.; Hunter, W. R. an introduction to the data for several metals. in handbook of optical constants of solids (ed. Palik, E. D.) **1998**, *3*, 245–247 (Academic Press in London).

(2) Lynch, D. W.; Hunter, W. R. comments on the optical constants of metals and an introduction to the data for several metals. in handbook of optical constants of solids (ed. Palik, E. D.) **1985**, *1*, 355–356 (Academic Press in London).

(3) Bennett, J. M.; Stanford, J. L.; Ashley, E. J. optical constants of silver sulfide tarnish films. *JOSA* **1970**, *60*, 224–232.

(4) Lynch, D. W.; Hunter, W. R. silicon dioxide (SiO2), type α (crystalline). in handbook of optical constants of solids (ed. Palik, E. D.) **1985**, *1*, 727 (Academic Press in London).

(5) Aboura, F. B.; Duché, D.; Simon, J. J.; Escoubas, L. ellipsometric study of the optical transitions of PC60BM and PC70BM thin films. *Chem. Phys.* **2015**, *450*, 102-108.
